# Cefiderocol and Intraventricular Colistin for Ventriculitis due to an Extensively Drug-Resistant *Pseudomonas Aeruginosa*

**DOI:** 10.2478/jccm-2024-0020

**Published:** 2024-04-30

**Authors:** João Melo e Silva, Diogo Oliveira, João A. Louro, Elisabete Monteiro

**Affiliations:** Hospital de Vila Franca de Xira, Lisboa, Portugal; Centro Hospitalar de Trás-os-Montes e Alto Douro, Vila Real, Portugal; Hospital Professor Doutor Fernando Fonseca EPE, Lisboa, Portugal; Centro Hospitalar Universitário de São João, Porto, Portugal

**Keywords:** cefiderocol, extensively drug-resistant, intraventricular therapy, *Pseudomonas Aeruginosa*, rheumatoid arthritis, ventriculitis

## Abstract

Rheumatoid arthritis, an inflammatory rheumatic disease predominantly affecting small limb joints, frequently compromises the cervical spine, resulting in spinal instability and the potential surgical necessity. This may result in severe complications, such as ventriculitis, often associated with a high mortality rate and multidrug-resistant organisms. A major challenge lies in achieving therapeutic antimicrobial concentrations in the central nervous system.

The authors present a case of a 65-year-old female, with cervical myelopathy due to severe rheumatoid arthritis. Following surgery, the patient developed ventriculitis caused by an extensively drug-resistant *Pseudomonas Aeruginosa*. Early diagnosis and prompt treatment played a crucial role in facilitating neurological and cognitive recovery.

## Introduction

Rheumatoid arthritis is an inflammatory rheumatic disease of autoimmune etiology, which manifests in the form of progressive, destructive, and deforming polyarthritis. It affects around 1% of the population, being women more affected (ratio 3:1), and its prevalence increases with age. It most often affects the small joints of the limbs. The cervical spine is also commonly compromised, resulting in spinal instability and, in severe cases, neurological impairment [1,2].

The surgical indication must be considered. One of the most feared complications is infection, especially considering the immunosuppression status of these patients. Possible infections include skin and soft tissue infection, osteomyelitis, meningitis, ventriculitis, brain abscess, endocarditis, and peritonitis [3].

The majority of infections occur in the first month and are mainly due to exogenous colonization from skin flora agents. Other possible etiologies are hematogenous or contiguity. In these cases, risk factors for multidrug-resistant (MDR) *Staphylococcus* species and MDR Gram-negative must be considered [4].

Ventriculitis is associated with high mortality, which can be improved with adequate initial treatment. Among other risk factors, the presence of a cerebral spinal fluid (CSF) leak is associated with an increased risk for ventriculitis [5,6]. Treatment of healthcare-associated ventriculitis and meningitis presents difficulties in obtaining therapeutic antimicrobial concentrations in the central nervous system (CNS). These complex infections are frequently due to MDR agents that have a poor response to intravenous antibiotics and can lead to inappropriate, discordant antibiotic treatment, which may adversely affect the outcome [5,7].

## Case presentation

We present a case of a 65-year-old female with a medical history of severe rheumatoid arthritis with cervical spondylosis myelopathy, previously submitted to cervical interventions (C1–C2 fixation, C3–C4 microdiscectomy, and C4–C5 fixation). She evolved with worsening of the spastic tetraparesis, due to C2–C5 severe kyphosis and sinking of the plate in C5. Consequently, a decision was made to pursue a new surgical intervention aimed at decompressing the spinal cord.

The surgical intervention was performed in two separate stages: corporectomy C3–C5, radical discectomies C2–C3 and C5–C6, arthrodesis C2–C6, fixation with anterior cervical plate, and posterolateral fusion. The surgeries occurred without major complications, although in the second stage intervention, there was an incidental laceration of the dura mater.

Given the anatomically challenging airway and pronounced laryngeal edema, a tracheostomy was deemed necessary to facilitate neurological evaluation, as well as to initiate early mobilization and rehabilitation. After the surgery, the emergence of transudate drainage at the surgical drain site raised concerns, and cervical magnetic resonance imaging (MRI) confirmed the presence of cerebrospinal fluid (CSF) fistulous paths.

Despite these intercurrences, the patient exhibited a favorable recovery during hospitalization. However, on the 22nd day, a significant clinical deterioration occurred. Neurological examination revealed a Glasgow coma scale of 14, marked by confusion and agitation. Pupils were isocoric and reactive to stimulus. There were no motor or sensitive deficits observed. Additionally, she had a fever with analytical studies indicating an elevation in infectious parameters (White blood count 17.630 cells/μL and C-reactive protein 21.7 mg/L), prompting an urgent computed tomography scan. A hydrocephalus with possible intraventricular purulent content was unveiled. Intravenous meropenem (2g 8/8h) and vancomycin (target steady-state *plateau* concentration of 20 to 25 mg/L) were promptly initiated, and an external ventricular drainage (EVD) system was placed.

**Fig. 1.**
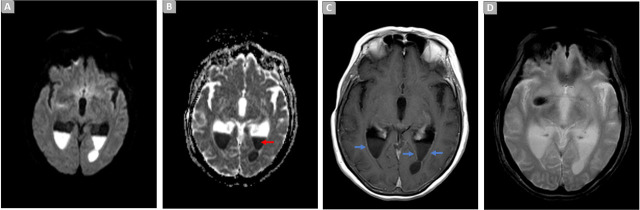
Brain MRI. Axial diffusion weighted image (A), ADC map (B), T1 SE post-contrast (C), and T2*-GRE (D), demonstrating signs of ventriculitis manifested by an increase in dimensions and ‘tension’ of the walls in the occipital and temporal horns of the ventricular system [C, blue arrows]. Content with restricted diffusion and a liquid level in the occipital horns: indicating purulent content [A–B: red arrow]. There is no significant blooming in T2* suggestive of hemorrhage [D].

To assess the extent of the central nervous system (CNS) infection, a cerebral and cervical MRI was conducted. The findings demonstrated signs of ventriculitis with a purulent collection (Figure 1), anterolateral CSF fistulous pathways through C3–C4, and epidural collections, suggestive of empyema.

The Neurosurgery team conducted a thorough evaluation and determined that, due to the extension of the infection, surgical source control was not feasible. Furthermore, the removal of the cervical prosthesis would result in severe spine instability.

The microbiological study revealed the presence of *Pseudomonas aeruginosa* extensively drug-resistant (XDR) in both CSF and in a bronchial respiratory sample (BRS). Accordingly, the antibiotic regimen was changed to intravenous ceftolozane/tazobactam (3g 8/8h) along with colistin (4.500.000 IU 12/12h), complemented by intraventricular therapy (IVT) with colistin (300.000 IU a day). Concurrently, the EVD system was replaced. Despite these interventions, there was no discernible clinical or analytical improvement in the subsequent days.

**Fig. 2.**
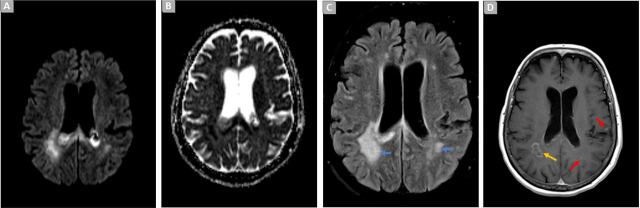
Brain MRI. Axial diffusion weighted image (A), ADC map (B), T2/FLAIR (fluid-attenuation-inversion-recovery) (C) and T1 SE post contrast (D), showing increased signal in T2iFLAIR image at the adjacent bilateral periventricular parenchyma [C, blue arrows], with restricted diffusion [A-B] and anomalous contrast enhancement [D], exhibiting signs of loculation in the right parietal white matter [D, yellow arrow] and some focal contralateral nodules [I), red arrows].

A follow-up microbiological study, conducted three weeks later, revealed the presence of a new strain of XDR *Pseudomonas aeruginosa* in a BRS, expressing in vitro resistance to Ceftolozane/Tazobactam (Table 1). MRI reassessment was suggestive of cerebritis with abscess formation in the right parietal region (Figure 2).

**Table 1.** Evolution of *Pseudomonas Aeruginosa* susceptibility testing.

**Antibiogram**	**CSF (day 25)**	**BRS (day 25)**	**BRS (day 48)**
Piperacillin-tazobactam	R	R	R
Amikacin	-	-	-
Aztreonam	R	R	R
Cefepime	R	R	SIE
Ceftazidime	R	R	R
Ciprofloxacin	R	R	R
Imipenem	R	R	R
Meropenem	R	R	R (>32 mg/l)
Tobramycin	S	S	S
Levofloxacin	R	R	R
Colistin	S	S (1.0 mg/l)	S (2.0 mg/l)
Ceftazidime-avibactam	R	R	R (>256 mg/l)
Ceftolozane-tazobactam	S	S	R
Imipenem-cilastin-relebactam	R (6.0 mg/l)	R	R (4.0 mg/l)
Meropenem-varobactam	-	-	-

Abbreviations: BRS- bronchial respiratory sample; CSF- cerebral spinal fluid; day 25/48 – 25/48 days after admission; R- resistant; S- susceptible; SIE- susceptible increased exposure.

Despite CSF not having any growth at this time, the clinical deterioration and the emergence of a resistant strain in the BRS led to an escalation to intravenous Cefiderocol (2g 8/8h), maintaining IVT Colistin.

Throughout hospitalization, sedation was gradually reduced to assess the neurological condition. In the first weeks, there were no signs of regaining consciousness, but the patient eventually demonstrated a slow and progressive awakening.

**Fig. 3.**
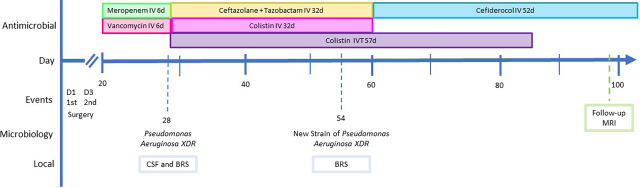
Timeline of events, microbiological findings, and antimicrobial periods. Abbreviations: BRS- bronchial respiratory sample: CSF- cerebral spinal fluid d- days: I, intravenous: IVT- intraventricular: Nal- Magnetic resonance imaging: XDR- Extensively drug-resistant.

Follow-up cranial MRI was not suggestive of infection worsening, and there was even attenuation of the signal in the areas of cerebritis. Considering the significant clinical and radiological improvement, the CNS infection was considered as treated, after completing 13 weeks of antibiotic therapy (Figure 3).

Throughout this period, there was a remarkable clinical improvement, with cognitive function recovery, including preserved orientation and the ability to collaborate in simple tasks. No memory deficits or motor or sensory changes were observed.

## Discussion

Rheumatoid arthritis stands as the most common inflammatory condition affecting the cervical spine and may potentially impact up to 80% of individuals. Surgical instrumentation for degenerative spine alterations is highly complex, carrying significant risks such as irreversible spinal cord damage or CNS infection [1].

According to the Assessment by the Review on Antimicrobial Resistance, projections indicate that up to 10 million individuals could die annually from antimicrobial resistance by the year 2050. The World Health Organization has classified carbapenem-resistant variants of *Enterobacterales*, *Pseudomonas aeruginosa*, *Acinetobacter baumannii*, and third-generation cephalosporin-resistant *Enterobacterales*, as “priority 1: critical” pathogens for which new treatments are urgently needed [8,9].

Within the healthcare environment, infections caused by XDR *Pseudomonas aeruginosa* strains carry significant morbidity and mortality. Indeed, *Pseudomonas aeruginosa* is one of the most frequently isolated and demanding pathogens, rarely eradicable, contributing to a high incidence of persistent and recurrent infections [10].

CSF cultures play a pivotal role in establishing the diagnosis of healthcare-associated ventriculitis and meningitis. Nonetheless, their sensitivity can be as low as 20% and 50% and may be diminished if antibiotics are administered before the sampling process [5]. In the case of healthcare-associated ventriculitis and meningitis, empirical antibiotic treatment is recommended with vancomycin in combination with an anti-pseudomonal beta-lactam, such as cefepime, ceftazidime, or meropenem [4,6].

The administration of antimicrobial therapy directly into the ventricle may become necessary for infections difficult to treat with intravenous therapy alone or when the patient is unable to bear a surgical procedure. However, IVT efficacy and safety have not been confirmed in controlled trials and the US Food and Drug Administration does not recommend their general use [6]. In 2017, the Infectious Diseases Society of America published clinical practice guidelines that supported IVT with antimicrobials in cases of healthcare-associated ventriculitis and meningitis who exhibit poor response to systemic therapy [4,6].

Since adopting this approach, some studies have shown that administering specific antibiotics into the ventricular system can achieve therapeutic concentrations at the infection site and lead to positive outcomes [7]. It's worth mentioning that the European Medicines Agency has approved the IVT use of colistin, at a dose of 300.000 IU daily, in patients with ventriculitis and meningitis due to MDR gram-negative bacteria or in those who failed therapy with intravenous agents [5].

Cefiderocol stands out as a new siderophore cephalosporin for the treatment of carbapenem-resistant gram-negative bacterial infections, including carbapenemase-resistant *Enterobacterales* and nonfermentative gram-negative *bacilli* such as *Acinetobacter spp, Pseudomonas spp,* and *Stenotrophomonas spp*. Cefiderocol enters using the bacteria's iron uptake system [11,12]. The European Medicines Agency has approved the administration of cefiderocol for patients with limited treatment options.

In 2020, Bavaro et al reported a patient with a neurosurgical site infection caused by XDR *Pseudomonas aeruginosa*, which was effectively treated with Cefiderocol [10]. Subsequently, a few cases of ventriculitis with XDR *Pseudomonas aeruginosa* treated with Cefiderocol have been reported [11,13].

While the ideal antimicrobial scheme and the duration of treatment remain to be determined, the management often involves neurosurgical intervention. Effectively managing infections typically demands a multidisciplinary team approach that includes treatment with antibiotics, debridement, and removal of associated devices [4,5].

## Conclusion

Cefiderocol emerges as an innovative antibiotic able to treat MDR bacteria *Pseudomonas aeruginosa* infection, even CNS infections. The use of IVT antibiotics may have the benefit of overcoming the blood-brain barrier.

The authors intend to draw attention to the principles of adequate antibiotic therapy to avoid the development of antimicrobial resistance.
